# Microbiological, sensory, and physicochemical quality of curd cheeses originating from direct sales

**DOI:** 10.14202/vetworld.2021.3038-3047

**Published:** 2021-11-29

**Authors:** Ewa Januś, Piotr Sablik, Małgorzata Jakubowska, Klaudia Wróbel

**Affiliations:** 1 Laboratory for Organic Production of Food of Animal Origin, Institute of Animal Breeding and Biodiversity Conservation, University of Life Sciences in Lublin, Poland; 2 Department of Ruminant Science, Faculty of Biotechnology and Animal Husbandry, West Pomeranian University of Technology, Szczecin, Poland; 3 Laboratory of Commodity Science of Food Products, Department of Microbiology and Biotechnology, Faculty of Biotechnology and Animal Husbandry, West Pomeranian University of Technology, Szczecin, Poland

**Keywords:** curd cheese, microbiology, physiochemistry, sensory quality

## Abstract

**Background and Aim:**

Curd cheeses are characteristic elements of the dairy assortment, mainly in Central and Eastern European countries, and constitute a numerous and diverse group of dairy products. The aim of the study was to assess the physicochemical, microbiological, and sensory quality of curd cheeses available in marketplaces in Lublin, where they were purchased through direct sales from producers.

**Materials and Methods:**

The research material was household-produced curd cheese purchased 4 times (at 2-week intervals) from three producers. The physicochemical parameters (i.e., the total protein and fat content, active acidity, and titratable acidity) were determined in the cheeses. Microbiological assays were performed to evaluate the total number of bacteria (on milk agar), the number of yeasts and molds (on Sabouraud medium), and the number of coliform bacteria (on MacConkey medium). A general sensory evaluation was performed by a five-person panel, who assessed the appearance and color, texture, flavor, and aroma of the samples.

**Results:**

The cheeses exhibited significant differences in their protein and fat contents, but these values were within the allowable limits. Most of the evaluated cheeses had normal levels of active and titratable acidity; substantially lower titratable acidity and higher pH values were detected only in the samples from supplier A. The total number of bacteria in the curd cheese samples was high (3.2×10^8^ colony-forming units [cfu]×g^-1^ cheese) and varied substantially (from 3.6×10^7^ to 8.6×10^8^ cfu×g^−1^). The growth of Gram-negative bacterial colonies on MacConkey medium was observed in the samples from suppliers B and C (5.5×10^3^ and 1.7×10^4^ cfu×g^−1^, respectively), which is an undesirable phenomenon. The number of colonies cultured on Sabouraud medium and identified as yeast-like microorganisms ranged from 1.8×10^4^ (product from supplier A) to 4.9×10^5^ cfu×g^−1^ (cheese from supplier C). The scores in the sensory evaluation of the tested curd cheeses were low. The highest mean scores were achieved for appearance and color (4.25-4.45 points). Conversely, flavor and aroma received the lowest score (3.17 points). The highest scores for both the overall assessment and each parameter separately were awarded to the curd cheese produced by supplier A.

**Conclusion:**

Our results suggest poor hygienic conditions during milk collection and processing, as well as during the distribution of these dairy products. Altogether, the purchase of products from direct sales may be associated with risks related to poor consumer health and food quality.

## Introduction

Milk and its products are one of the basic foods in the human diet; many populations could not balance their diet without dairy products [1]. A numerous and diverse group of dairy products are curd cheeses, which are characteristic elements of the dairy assortment, mainly in Central and Eastern European countries [2]. They are consumed eagerly, and their attractiveness is associated with their availability, a long-standing tradition of consumption, established eating habits, rich assortment, and relatively low prices [3,4]. Curd cheeses have been categorized as value added products due to their positive effect on health (i.e., a reduction of the risk of various diseases, such as cancer, HIV, and osteoporosis) and improvement of the overall health status in humans [1]. In Poland, the consumption of curd cheeses has exceeded the consumption of ripened and processed cheeses for many years [5]. As shown in the Central Statistical Office data [6] in Poland, the monthly consumption of cheese and curd cheese in 2017 was 0.86 kg/person.

As reported by Verraes *et al*. [7], consumers’ attitudes indicate a trend toward the increased consumption of unprocessed or minimally-processed food (e.g., raw milk and raw milk products). Foods such as curd cheese, butter, or cream can be purchased through direct sales from the producer, as the provisions of the European Union food law allow this type of sale of animal and vegetable products in their Member States. As specified by the Regulation of the Minister of Agriculture and Rural Development on the maximum amount of food sold through agricultural direct sales, as well as the range and methods for documentation thereof [8], farm producers can sell a maximum of 52 thousand liters of raw milk or raw milk and colostrum annually in total, 10.4 thousand liters of raw cream, and 2.6 thousand kilograms of milk- or colostrum-based products in total.

The course of complex technological processes applied in the production of cheese, and hence the nutritional value and broad-sense quality of the product, are largely dependent on the quality of the milk [9]. The technological quality of milk depends mainly on the protein content, especially casein, the amount of fat, and the dispersion of fat globules. Fat dispersion determines the texture, aroma, and physicochemical properties of cheeses [10,11]. Good quality curd cheeses are produced by the process of acidic casein coagulation, resulting from targeted fermentation, with the use of lactic acid bacteria (LAB) added as leaven or starter cultures [12]. Therefore, lactic acid fermentation is the most important process in the production of curd cheeses, as it determines the quality of the curd and the course of further production procedures [13]. An additional important factor in the production of high-quality curd cheeses is the use of raw materials with excellent hygienic quality. Microbiological contamination of milk during the collection, storage, and distribution processes (e.g., the presence of pathogenic microflora) may significantly affect the quality and sensory value of curd cheeses [14,15]. The resistance of *Escherichia coli* bacteria to environmental conditions (low temperature, pH≤3) is responsible for the presence of these bacteria in marketed curd cheeses, which reduces their quality (cheese swelling) [15]. *Pseudomonas aeruginosa* is another pathogenic Gram-negative bacteria often detected in these products [16]. The porous structure of curd cheeses supports the growth of relatively anaerobic yeasts as well as molds, both on the surface and inside the cheese. As reported by Ziajka [16], dangerous aflatoxin may be produced in curd cheeses contaminated with *Aspergillus flavus* after 9 days of storage at a temperature exceeding 18°C. Therefore, it is extremely important to educate milk producers of this risk and implement appropriate procedures to minimize the risk of food poisoning; these measures include the application of good agricultural practices and good hygienic practices at all stages of production [17].

The disadvantage of products purchased through direct sales is their several-day durability [18]; this is associated with the composition of the product as well as the type of packaging, which does not always provide sufficient protection from the external environment, microbiological and chemical contamination, or mechanical damage [19]. Appropriate vacuum-sealing techniques are not normally used in direct sales.

In Poland, milk intended for the dairy industry is assessed in the dairy plant for compliance with applicable standards. In contrast, milk and dairy products (e.g., cream, butter, curd cheese) available in street markets and bazaars are not subject to any quality control; such milk and its products are sold in packaging and under conditions that do not meet the appropriate hygienic standards. Nevertheless, these products are very popular and purchased eagerly. According to the common consumers’ view, they have better nutritional value and are “healthier” than products sold by retailers. However, it should be emphasized that the production and distribution conditions of this milk and its products raise concerns about their quality, in terms of consumer health, and their suitability for consumption [20].

The aim of the study was to assess the microbiological, physicochemical, and sensory quality of curd cheeses available in marketplaces in Lublin and sold directly from producers. The quality of artisanal cheeses has not often been a frequent subject of scientific analysis; therefore, we considered it necessary to investigate this topic, particularly given its great importance for the consumers and purchasers of artisanal cheese products.

## Materials and Methods

### Ethical approval

Ethical approval was not needed for this study.

### Study period and location

The study was conducted from April to September 2019. The study material consisted of curd cheeses produced using household methods (gradual heating of non-standardized and non-pasteurized sour milk to a temperature of 40-50°C, cooling, and straining the curd). The curd cheeses were purchased directly from three producers (designated for the analysis as suppliers A, B, and C) in marketplaces located in Lublin in April–May 2019. The sellers declared that the cheeses were produced in the evening hours on the day preceding the sale. The producers used milk from cows showing no symptoms of inflammation or other udder diseases and added no preservatives or starter cultures to modify the natural course of milk fermentation during the production process. Approximately 1 kg of curd cheese in four portions was purchased from each seller at 2-week intervals. After the purchase, the cheese samples were packed, labeled, and transported to the microbiological laboratory in insulated thermal bags. The curd cheeses were analyzed no later than 2 h after their purchase.

### Methods

The analyses consisted of an evaluation of the microbiological and physicochemical (pH, titratable acidity, protein, and fat content) parameters and a sensory assessment of the products.

#### Microbiological analysis

The microbiological tests evaluated the total bacterial count (TBC, on milk agar) in accordance with PN-EN ISO 4833:2003 [21], the number of yeast and mold cells (on Sabouraud medium) in accordance with PN-ISO 6611:2007 [22], and coliform bacteria (on MacConkey medium) in accordance with PN-93/A-86034-08:1993 [23]. Ten grams of cheese were first homogenized in 90 mL of sodium citrate solution for 1 min. Subsequent serial dilutions were made in Ringer’s solution. Inoculations of ten-fold dilutions of the curd cheese samples were performed with the standard deep inoculation method. The TBC was determined by inoculation of the suspension of microorganisms on milk agar medium; these microorganisms were cultured at a temperature of 30 °C for 72 h. Fungi were isolated in Sabouard’s medium in cultures at 25°C for 3-5 days, whereas coliform bacteria were grown in MacConkey’s medium at 37°C for 24 h. After incubation under the specified conditions, colonies were counted and counts were converted into the number of colony-forming units (cfu) per 1 g of product.

#### Physicochemical analysis

The pH was measured with a glass combination electrode using an Elmetron pH-meter. The potential acidity of the curd cheeses expressed in Soxhlet-Henkel degrees [24] was determined at room temperature; we used the arithmetic mean of two parallel measurements as a result. The Kjeldahl method was used to determine the total protein content, and the Gerber butyrometric method was used to determine the fat content [24].

#### Sensory evaluation

Sensory evaluation was conducted in a well-lit room free of foreign odors and at room temperature. It was performed by a panel of five people who had been trained in the assessment methodology before starting the evaluation. Each person occupied a separate place in the room to prevent mutual communication. The panelists received appropriately labeled 20-g samples of each curd cheese and assessed each sample’s appearance and color, texture, flavor, and aroma on a scale of 1 to 5 (where 1 - the lowest score and 5 - the highest score). The criteria for sensory evaluation were developed based on the methodology proposed by Pieczonka [25]; Table-1. Next, after accounting for the appropriate weighting factors (Table-1), an overall score for the curd cheese samples was calculated.

**Table-1:** Scores of curd cheeses in a 5-point scale [25].

Scores	Quality parameter (weighting factor)		

	Appearance and colour (0.15)	Texture (0.25)	Flavour and aroma (0.60)
5 very good	White to slightly creamy color; homogeneous	Homogeneous, compact, with no lumps	Pure, mild, aromatic, slightly acidic
4 good	White to slightly creamy color; non-homogeneous	Homogeneous, loose, finely grained, acceptable lumps	Acidic, acceptable, slightly unclear after taste
3 satisfactory	White to slightly creamy color; non-homogeneous	Lumpy, slightly crumbly, slightly hard	Excessively sour, foul aftertaste
2 unsatisfactory	Excessively yellow color of the entire mass	Crumbly, slightly slimy	Vinegar-acidic, stinging, irritating, slightly moldy
1 unacceptable	non-homogeneous color, grey tone	Slimy, crumbly, grainy, rubbery	Bitter, flavorless, bland, yeast-like, musty, moldy

### Statistical analysis

The results of the microbiological, physicochemical, and sensory evaluation of the curd cheeses were summarized in Excel. Statistical analysis was performed using Statistica ver. 13.1 (StatSoft Inc., Poland). The Duncan test was used to assess the significance of differences between the means, whereas the χ^2^ test was used to assess the frequency of various scores granted to the different cheeses during the sensory evaluation. A significance level of p≤0.05 was used when no significance of differences was found at p≤0.01.

## Results and Discussion

The evaluated cheeses were characterized by a high total number of bacteria (Table-2), with an average of 3.2×10^8^ cfu in 1 g of cheese. The number of bacterial cells varied between the samples, ranging widely from 36×10^6^ to 860×10^6^ cfu×g^−1^. Berthold and Stachura [26] found that the total number of microorganisms in organic curd cheese ranged from 2.5×10^5^ to 6.8×10^10^, and each cheese sample exhibited bacteria that constituted impurities derived from the production process (re-infection). In their study, only 6% of samples were characterized by a TBC >10^5^ to 10^6^ cfu×g^−1^ of cheese. In as many as 76% of the samples, the number of microorganisms that demonstrated improper hygiene of the technological process ranged from >10^4^ to 10^7^ cfu/g [26]. As concluded by Knysz *et al*. [27], cheeses produced on small farms using traditional methods with no pasteurization process or highly advanced technologies have a highly variable microbiological quality and nutritional value.

**Table-2:** Numbers of microorganisms detected in the curd cheese samples.

Group of microorganisms	Supplier	Number of analyzed samples	Numbers of microorganisms			

			X̄	SD	Min.	Max.
Total bacterial count	A	16	3.2×10^8^	2.2×10^8^	3.6×10^7^	7.0×10^8^
	B	16	4.1×10^8a^	1.9×10^8^	1.0×10^8^	8.0×10^8^
	C	16	2.3×10^8b^	2.2×10^8^	7.0×10^7^	8.6×10^8^
	Σ and X̄	48	3.2×10^8^	2.2×10^8^	3.6×10^7^	8.6×10^8^
*Coliforms*	A	16	no growth^A^	no growth	no growth	no growth
	B	16	5.5×10^3a^	1.0×10^4^	1.0×10^3^	4.0×10^4^
	C	16	1.7×10^4Bb^	2.3×10^4^	4.0×10^3^	8.0×10^4^
	Σ and X̄	48	7.5×10^3^	1.6×10^4^	no growth	8.0×10^4^
Yeast and molds	A	16	1.8×10^4A^	3.2×10^4^	5.0×10^3^	8.0×10^4^
	B	16	2.7×10^5^	4.2×10^5^	1.0×10^4^	1.4×10^6^
	C	16	4.9×10^5B^	4.8×10^5^	1.0×10^4^	1.7×10^6^
	Σ and X̄	48	2.6×10^5^	4.1×10^5^	5.0×10^3^	1.7×10^6^

Means marked with different letters differ significantly: capital letters - for p≤0.01; lowercase letters - for p≤0.05

In our study, there were significant differences in the total number of bacteria in the tested curd cheeses. The lowest number (i.e., on average, 2.3×10^8^ cfu×g^−1^) was detected in product from supplier C; this value differed (p=0.0248) from that calculated for the cheese from supplier B, which contained, on average, 4.1×10^8^ cfu×g^−1^. The average TBC (3.2×10^8^ cfu×g^−1^) in the curd cheese from supplier A had an intermediate value and did not differ statistically from that of the cheese from suppliers B and C.

**Figure-1:**
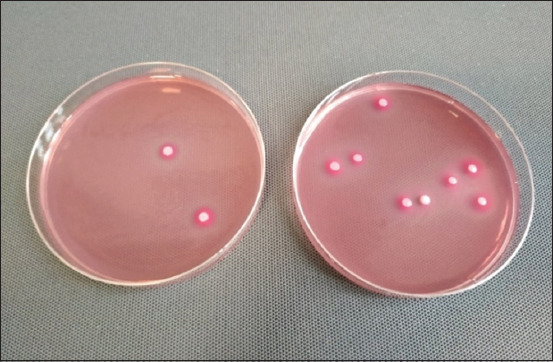
Colonies of bacteria present in curd cheese samples cultured on MacConkey medium (*own resources*).

For the curd cheese samples from suppliers B and C, the growth of Gram-negative bacteria on MacConkey medium was 5.5×10^3^ and 17.1×10^3^ cfu×g^−1^, respectively; these values were statistically different (p=0.0385). Gram-negative bacteria were not detected in the curd cheese from supplier A. The colonies grown in the curd cheese samples from suppliers B and C were convex, round, pink, and glossy with a pink coating (Figure-1), which suggest the presence of coliform bacteria. At present, the microbiological quality of food products is specified by Commission Regulation (EC) No. 2073/2005 of November 15, 2005, on microbiological criteria for foodstuffs [28]. This regulation establishes microbiological criteria for cheeses. In the case of cheese made from raw milk, the regulation only specifies that the content of coagulase-positive bacteria should be below 10^5^ cfu×g^−1^. In the case of cheeses produced from heat-treated milk, <10^3^ cfu×g^−1^ of *E. coli* bacteria should be present. Hence, the results obtained in this study can be related back to the microbiological criteria contained in the Polish Standard PN-91/A-86300 [29], which recommend that no coliform bacteria be present in 0.001 g of product; this indicates that the health safety criterion was not met for the cheeses from suppliers B and C.

In the study conducted by Bis and Mędrela-Kuder [20], coliform bacteria were detected in as many as 72% of artisan cheese samples, and fecal streptococci were found in 75% of the samples. The presence of these microorganisms should be considered negative, as these bacteria are part of an undesirable microflora in food products, indicating potential fecal contamination, and pose a health risk to the consumer [30]. Their presence is also undesirable as it deteriorates the technological quality of the product, causing the early swelling and cracking of cheeses, as well as alterations to the flavor and aroma [20,26,31]. This pattern was confirmed in the present sensory evaluation results: the curd cheeses were characterized by an excessively sour and foul aftertaste (Tables-1-4).

**Table-3:** Changes in the numbers of microorganisms in the curd cheese samples from each supplier in consecutive studies.

Group of microorganisms	Number of analysis	Number of analyzed samples	Supplier A		Supplier B		Supplier C	
		
			X̄	SD	X̄	SD	X̄	SD
Total bacterial count	1	4	1.1×10^8A^	5.5×10^7^	4.9×10^8^	4.9×10^7^	1.1×10^8a^	5.0×10^7^
	2	4	3.6×10^8^	2.7×10^8^	5.4×10^8^	2.1×10^8^	9.8×10^7a^	1.3×10^7^
	3	4	3.1×10^8^	1.3×10^8^	3.7×10^8^	2.5×10^8^	4.4×10^8b^	3.5×10^8^
	4	4	5.2×10^8B^	1.8×10^8^	2.6×10^8^	9.5×10^7^	2.9×10^8^	1.4×10^8^
	Σ and X̄	16	3.2×10^8^	2.1×10^8^	4.1×10^8^	1.9×10^8^	2.3×10^8^	2.2×10^8^
*Coliforms*	1	4	no growth	-	1.7×10^4a^	1.5×10^4^	9.3×10^3A^	7.8×10^3^
	2	4	no growth	-	no growth^b^	-	no growth^A^	-
	3	4	no growth	-	3.3×10^3b^	3.8×10^3^	1.3×10^4A^	4.9×10^3^
	4	4	no growth	-	1.8×10^3b^	2.1×10^3^	4.7×10^4B^	2.9×10^4^
	Σ and X̄	16	no growth	-	5.5×10^3^	1.0×10^4^	1.7×10^4^	2.3×10^4^
Yeast and molds	1	4	7.0×10^4^ ^A^	1.0×10	1.7×10^5A^	4.3×10^4^	3.4×10^5a^	6.2×10^4^
	2	4	no growth^B^	^4^	5.9×10^4A^	4.1×10^4^	5.5×10^5^	9.0×10^4^
	3	4	no growth^B^	-	2.4×10^4A^	1.6×10^4^	6.6×10^4A^	4.5×10^4^
	4	4	no growth^B^	-	8.2×10^5B^	5.5×10^5^	1.0×10^6Bb^	7.0×10^5^
	Σ and X̄	16	1.7×10^4^	3.2×10^4^	2.7×10^5^	4.2×10^5^	4.9×10^5^	4.8×10^5^

Means marked with different letters differ significantly: capital letters - for p*≤*0.01; lowercase letters - for p*≤*0.05

**Table-4:** Results of the organoleptic evaluation of curd cheeses.

Parameter	Supplier	Number of evaluated samples	Scores			

			X̄	SD	Min.	Max.
Appearance and color	A	20	4.45	0.83	3	5
	B	20	4.35	0.49	4	5
	C	20	4.25	0.64	3	5
	Σ and X̄	60	4.35	0.66	3	5
Texture	A	20	3.95^A^	0.83	3	5
	B	20	2.75^B^	0.55	2	4
	C	20	3.35^c^	0.99	2	5
	Σ and X̄	60	3.35	0.94	2	5
Flavor and aroma	A	20	3.40	0.99	2	5
	B	20	3.05	0.69	2	4
	C	20	3.05	0.83	2	5
	Σ and X̄	60	3.17	0.85	2	5
Total scores	A	20	3.70^a^	0.83	2.40	5.00
	B	20	3.17^b^	0.51	2.45	4.15
	C	20	3.31	0.71	2.40	5.00
	Σ and X̄	60	3.39	0.72	2.40	5.00

Means marked with different letters differ significantly: capital letters - for p≤0.01; lowercase letters - for p≤0.05

**Figure-2:**
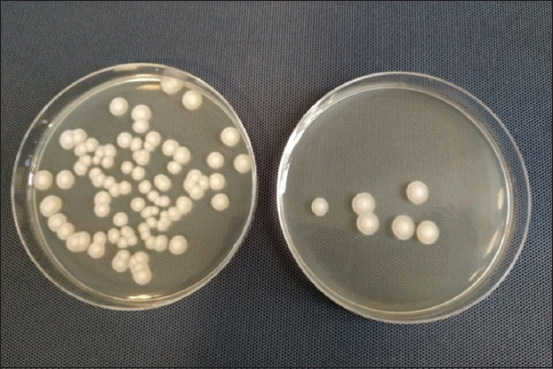
Colonies of microorganisms present in curd cheese samples cultured on Sabouraud medium (*own resources*).

The number of microorganisms grown on Sabouraud medium ranged from 1.8×10^4^ cfu×g^−1^ (curd cheese from supplier A) to 4.9×10^5^ cfu×g^−1^ (curd cheese from supplier C). The morphology of the colonies revealed that they were yeast-like microorganisms; we observed convex mucoid colonies with a diameter of approx. 2 mm and a yeast odor (Figure 2). The curd cheeses contained a higher amount of yeasts (10 000 cfu/g) than the quantity permitted by the Polish Standard (PN-91/A-86300) [29]. As shown by Urarte *et al*. [32], the presence of over 10^5^ cfu/g of yeasts worsens cheese quality. Infection of products with yeasts may be evidence of poor hygiene during milk collection and processing or may be related to fermentation (e.g., after the application of kefir cultures to the milk to produce the curd faster during the household cheese production process). Mold was not detected in the analyzed curd cheese samples.

In many cases, the microbiological assays of the curd cheeses were statistically significant differences between the suppliers (Table-3). Evident and quite regular changes were noted in the case of the curd cheese from supplier A (i.e., the TBC usually increased in the consecutive analyses [with the exception of analysis 3]). The difference between the first and fourth analysis terms was statistically significant (p≤0.01). No Gram-negative bacteria were detected in any of the curd cheese samples from supplier A, and yeast-like fungi were detected only in the first cheese batch. The highest TBC value in the curd cheese from supplier B (5.3×10^8^ cfu×g^−1^) was recorded during analysis 2; however, there were no differences between the mean values calculated in the subsequent analysis terms. Yeast was detected in all samples of curd cheese from supplier B, which is a negative finding because yeasts presence may be evidence of poor hygiene during milk collection and/or processing [32]. In the fourth analysis, the amount of yeast was higher, such that the mean value differed significantly (p≤0.01) from the other means. The results of the cultures growing on MacConkey medium showed that the curd cheese was only free of coliform bacteria in analysis 2. The curd cheese from supplier C had significant differences in the TBC range, and the means in the first and second analyses differed significantly from that of the third analysis. As in the case of the curd cheese from supplier B, yeast was detected in all analyses of this product, and coliform bacteria were only absent from the second analysis. In addition, the mean number of yeast colonies and coliform bacteria in the curd cheese from supplier C was higher than that in the samples from supplier B.

The organoleptic assessments of the curd cheeses (i.e., the scores for each feature separately and the overall rating) were not high (Table-4). The highest mean scores were recorded for appearance and color, at 4.25 and 4.45, respectively. In turn, flavor and aroma received the lowest scores, with the mean score for these parameters being only 3.17, with fluctuations from 3.05 to 3.40. The highest scores in the overall assessment as well as for each parameter separately were for the curd cheese from supplier A, whereas the lowest scores were for curd cheese from supplier B. Differences in the assessment of the flavor and aroma of the examined cheeses may have resulted from the different chemical composition of the milk used for their production. The lowest scores for flavor and aroma were awarded to the cheeses from suppliers B and C, which had a lower fat content (Table-5), a factor known to be a flavor carrier and responsible for sensory acceptability [33]. There were also differences in the texture (p≤0.01) and overall evaluation (p≤0.05) of the curd cheeses. Investigations conducted by Dmytrów *et al*. [19] demonstrated highly similar scores granted by the panelists for various sensory parameters of curd cheese, with the biggest differences being in the assessment of structure and texture. The observed differences in consistency may be associated with the different production processes used by the individual suppliers. Polish traditional curd cheeses are most often produced on small family farms from unpasteurized cow’s milk, in accordance with an old recipe [27]. The simplified manufacturing process of these products excludes the use of advanced technological solutions and production standards, which indicates that products from different manufacturers may exhibit a wide variety of quality traits. This phenomenon was confirmed by Siemianowski and Szpendowski [34], who found that, depending on the parameters of curd processing, the methods for the separation of curd grains and the further processing of separated curd mass, products may differ in basic chemical composition and texture. Moreover, the protein and fat content in milk have a considerable impact on cheese texture. As shown by Dmytrów [13], increased fat content in milk improves the plasticity and homogeneity of cheese mass. In turn, as suggested by Lucey *et al*. [35], cheese texture properties are largely dependent on the protein content; this is consistent with the results reported by Siemianowski *et al*. [36], who analyzed the effect of increasing the dry matter content on the texture of curd cheeses. Siemianowski *et al*. [36] showed that the hardness and adhesiveness of curds increased as the protein content increased. In the present study, these relationships were indirectly reflected in the results of the sensory evaluation of the curds; the curds from supplier A, whose cheeses had the highest protein and fat content, were characterized by a homogeneous and firm consistency.

The frequency of the scores for the analyzed characteristics of the curd cheeses is presented in Table-6. These data show that the appearance and color of the curd cheeses were most often judged to be a 4 or 5 (45.0% of the ratings each). The frequency of the different scores for this appearance and color depended on the supplier (p≤0.05). The product from supplier A more frequently 65.0% received a score of 5 points for appearance and color, whereas the curd cheeses from suppliers B and C most frequently received 4 points (65.0% and 55.0%, respectively). Nonetheless, we emphasize that the appearance and color of all curd cheeses were highly accepted, as these parameters were never rated as 1 or 2 points.

**Table-5:** Content of basic components and acidity of curd cheeses purchased from each supplier in consecutive analyses.

Parameter	Number of analysis	Number of analyzed samples	Supplier A		Supplier B		Supplier C	
		
			X̄	SD	X̄	SD	X̄	SD
Fat content (%)	1	4	12.41	0.94	11.32	0.68	11.87	0.79
	2	4	12.74^a^	0.87	10.62^b^	0.94	11.09	0.84
	3	4	11.91^a^	0.78	11.27	0.87	10.67^b^	0.64
	4	4	12.36^a^	0.86	10.95^b^	0.59	11.62	0.68
	Σ and X̄	16	12.36^a^	0.86	11.04^b^	0.77	11.31	0.74
Protein content (%)	1	4	16.48^a^	0.89	15.26^b^	1.03	15.36	1.01
	2	4	15.98^a^	0.87	14.97^b^	1.00	15.11	0.99
	3	4	16.79^Aa^	1.01	14.83^B^	0.94	14.96^b^	1.15
	4	4	15.60	1.24	15.03	1.21	15.28	1.06
	Σ and X̄	16	16.21^a^	1.00	15.02^b^	1.04	15.18^b^	1.05
Titratable acidity (°SH)	1	4	63.23^a^	1.03	75.02^b^	1.13	71.86^b^	0.86
	2	4	67.11^Aa^	30.89	78.56^B^	0.84	73.08^b^	1.21
	3	4	66.40^A^	0.74	78.89^Ba^	1.09	71.28^Bb^	1.18
	4	4	56.24^Aa^	0.98	80.66^B^	1.04	73.98^b^	0.94
	Σ and X̄	16	63.25^A^	0.91	78.28^Ba^	1.02	72.55^Bb^	1.05
Active acidity (pH)	1	4	4.71^Aa^	0.09	4.46^B^	0.09	4.51^b^	0.05
	2	4	4.68^a^	0.14	4.38^b^	0.04	4.48	0.07
	3	4	4.69^a^	0.08	4.37^b^	0.07	4.52	0.15
	4	4	4.74^Aa^	0.11	4.34^B^	0.12	4.47^b^	0.14
	Σ and X̄	16	4.71^Aa^	0.11	4.39^B^	0.08	4.50^b^	0.10

Means in rows marked with different letters differ significantly: capital letters - for p≤0.01; lowercase letters - for p≤0.05

**Table-6:** Frequency of scores given by the panelists in organoleptic evaluation of the curd cheese samples.

Parameter	Score (pts)	Supplier A	Supplier B	Supplier C	Total	ϰ**^2^ value**

		Number (%) of scores				
Appearance and color	1	-	-	-	-	12.89*
	2	-	-	-	-	
	3	4 (20.0)	-	2 (10.0)	6 (10.0)	
	4	3 (15.0)	13 (65.0)	11 (55.0)	27 (45.0)	
	5	13 (65.0)	7 (35.0)	7 (35.0)	27 (45.0)	
Texture	1	-	-	-	-	18.12**
	2	-	6 (30.0)	4 (20.0)	10 (16.7)	
	3	7 (35.0)	13 (65.0)	8 (40.0)	28 (46.6)	
	4	7 (35.0)	1 (5.0)	5 (25.0)	13 (21.7)	
	5	6 (30.0)	-	3 (15.0)	9 (15.0)	
Flavour and aroma	1	-	-	-	-	4.98
	2	4 (20.0)	4 (20.0)	5 (25.0)	13 (21.7	
	3	7 (35.0)	11 (55.0)	10 (50.0)	28 (46.6)	
	4	6 (30.0)	5 (25.0)	4 (20.0)	15 (25.0)	
	5	3 (15.0)	-	1 (5.0)	4 (6.7)	
Total scores	1 do<2	-	-	-	-	8.96
	2 do<3	4 (20.0)	9 (45.0)	7 (35.0)	20 (33.3)	
	3 do<4	7 (35.0)	10 (50.0)	8 (40.0)	25 (41.7)	
	4 do<5	7 (35.0)	1 (5.0)	4 (20.0)	12 (20.0)	
	5	2 (10.0)	-	1 (5.0)	3 (5.0)	


ϰ^2^ value significant at:

** p≤0.01;

* p≤0.05

There was a significant (p≤0.01) effect of the curd cheese producer on the frequency of different ratings for the texture parameter. In 28 cases (46.6%), this parameter was given 3 points; in the case of products from suppliers B and C, a score of 3 was the most frequently given score for texture (65.0% and 40.0% of the scores, respectively). The texture of the curd cheese from supplier A was rated at 3 and 4 points with the same frequency (35.0% each), but was given a score of 5 points in 30.0% of the scores. The curd cheese from supplier C received a score of 5 in only 15.0% of the ratings, and the product from supplier B was never rated 5 points for the texture. The good texture of the curd cheese from supplier A is also supported by the fact that this parameter was never given a score lower than 3 points. In turn, the curd cheeses from suppliers C and B were given a score of 2 points in 20.0 and 30% of cases, respectively.

Flavor and aroma score frequencies did not differ between the cheeses, with scores of 3 points accounting for the highest percentage of scores for the curd cheeses from each supplier, indicating a low consumer acceptance. The highest number of 3-point scores was granted to the curd cheese from supplier B (55.0%) and supplier C (50.0%), whereas a lower proportion of this score (i.e., 35.0%) was recorded in the evaluation of curd cheese from supplier A. Curd cheese from supplier A received the highest scores for flavor and aroma from the panelists, who rated it 4 (30.0%) and 5 points (15.0%) more often than the other products. The formation of the flavor and aroma of both fresh and ripened cheese is a very complex process [37]; these parameters are determined by the LAB strains used, the course of fermentation, and modification of enzymatic milk components (e.g., the effect on lactose, lipids, and proteins). Proteolysis has an impact on texture and leads to the formation of flavor peptides and free amino acids that form the precursors of aromatic compounds, such as diacetyl and acetaldehyde, whose presence may differentiate curd cheeses in terms of flavor and aroma [38]. Excessive acidification of milk (pH<4.4) is responsible for worse organoleptic properties in the finished product, as the cheese can be more greasy and sour. As shown by data in the literature, the appropriate pH of curd cheese should be between 4.4 and 4.6 [16]. In the present study, the average pH of the curd cheeses from individual suppliers ranged from 4.39 to 4.71, and likely did not have a large impact on the detection of a sour flavor by the panelists. Hence, we assume that the low flavor and aroma scores for the tested cheeses may have been caused by the abundance of coliform bacteria and yeasts; the presence of these microorganisms is responsible for altering flavor and aroma [31].

The overall sensory score of the curd cheeses often ranged from 3 to <4 points; the total percentage of such scores was 41.7%, and the proportion for individual products was 35.0, 50.0, and 40.0% in the case of the curd cheeses from supplier A, B, and C, respectively. The percentage of scores ranging from 2 to <3 points for the curd cheeses from suppliers B and C was higher (45.0% and 35.0%, respectively). The product from supplier A achieved the highest number of overall scores of 5, indicating the highest sensory acceptance.

Table-5 shows the basic chemical components and acidity of the curd cheeses assessed in the consecutive analyses. The total protein content in the products ranged from 14.83 to 16.79. Curd cheese from supplier A had a higher (p≤0.05) mean protein level (16.21%) than that of the products from suppliers B (15.02%) and C (15.18%). As demonstrated by data from the literature, the average protein content in curd cheese may range from 12 to 20% [16,39]. In turn, Litwińczuk *et al*. [40] have reported that the content of protein in low-fat and high-fat curd cheeses is 18 and 14 g/100 g of product, respectively. All the curd cheese samples analyzed in the present study contained a standard amount of protein.

The fat content in the analyzed curd cheeses differed (Table-5). Samples from supplier A had the highest average fat content in dry matter (12.36%), whereas the fat content in the samples from suppliers B and C was 11.04 and 11.31%, respectively. According to the PN-91/A-86300 recommendations [29], the fat content in full-fat, high-fat, and semi-fat curd cheeses should range between 42 and 15±2%; however, no content is specified by the standard for low-fat curd cheeses. As specified by the Codex General Standard for Cheese 283-1978 included in the Codex Alimentarius [41], cheese with fat content in dry matter equal to or >10% but lower than 25% can be classified as partially skimmed. Hence, all the curd cheese samples analyzed in the present study can be classified as low-fat or partially-skimmed cheeses. It is important to note that there are no recommendations specifying the standard amount of fat and protein in curd cheeses from household production. Differences in the fat and protein content of the curd cheeses analyzed in this study may have resulted from the simplified production process, which excludes the use of advanced technological solutions or compliance with production standards [27]. Importantly, the protein and fat content in the final product is determined by the concentration of these components in the milk intended to be used for the production of curd cheese [42].

The active acidity of the curd cheese purchased for the four analyses was in the range of 4.39-4.71, with pH values differing between the evaluated products (Table-5). As shown in the literature data, the proper pH value of curd cheese should range between 4.4 and 4.6 [16,36,43]; these pH values indicate an appropriate process of milk acidification until achievement of the isoelectric point of milk proteins [44]. In the present study, only the curd cheeses from suppliers B and C met these requirements (pH=4.39-4.51), whereas the samples from supplier A were characterized by lower acidity (pH=4.71). In investigations of the impact of the packaging system on the quality of curd cheese produced with various methods, Pluta *et al*. [45] noted a slight decrease in the pH value and an increase in titratable acidity in cheeses; they suggested that the magnitude of these changes depended on the production system. Hence, we can assume that differences in the production process of the curd cheeses analyzed in the present study had an impact on their pH value [46]. The titratable acidity of the curd cheeses was in the range of 63.25 to 78.28°SH. Regardless of the term of analysis, the lowest titratable acidity was recorded in the product from supplier A (p≤0.01; Table-5). Our titratable acidity values are consistent with the Polish Standard [29], which specifies that it should not exceed 100–110^o^SH for semi-fat and low-fat curd cheeses. As indicated by Siemianowski *et al*. [36] after Kolanowski, the acidity of curd cheeses should not exceed 80-110^o^SH. The lower acidity noted in the curd cheese from supplier A may be associated with the lower degree of acidification of the raw material used for production. Presumably, differences in the acidity of the products may have been related to the chemical composition of milk and differences in their buffering capacity. The acidity of curd cheeses is also influenced by the course of lactic acid fermentation, which depends on the nutritional requirements of the LAB used to acidify the milk [47].

## Conclusion

The results obtained in this study should be considered monitoring data; they are an important preliminary step in extensive research involving a larger number of producers and consisting of the detailed determination of parameters that are essential for the consumers of food produced by small agricultural manufacturers. This type of food is gaining popularity and is increasingly being sought on the food market.

As shown by the analyses in the present study, the evaluated curd cheeses from the individual suppliers had a high mean total number of bacteria. The detailed microbiological analyses of samples provided by two suppliers revealed the presence of Gram-negative coliform bacteria, which constitute a negative, undesirable microflora in food products; their presence may indicate re-infection of the analyzed products during processing and distribution. Some microorganisms can pose a threat to consumers’ health. The presence of yeast-like microorganisms in the curd cheeses may imply a low level of hygiene during the milk collection and production processes. In addition, the overall sensory evaluation of the curd cheeses was low. Appearance and color had the highest score, whereas flavor and aroma were given the lowest rating; this was probably related to the presence of coliform bacteria and yeasts. The active acidity of the curd cheeses at the level of 4.39-4.71 pH should be regarded as a desirable level, indicating a normal acidification process. The protein content in the examined cheeses was also within the normal range. The fat content classified the products as low-fat or semi-fat curd cheeses.

The results of the microbiological analyses and sensory evaluation of the artisanal curd cheeses available for direct sale indicate that the purchase of such products may pose a risk due to their low food and health quality. The results also suggest the need to improve the producers’ sanitary practices during milk collection and processing and during the storage and distribution of the finished products.

## Authors’ Contributions

EJ: Designed the concept of research, coordinated the collection of the samples, performed the statistical analysis, and drafted the manuscript. PS: Designed the concept of research, analyzed and interpreted data, and drafted and revised the manuscript. MJ: Designed the concept of research, performed the statistical analysis, and drafted and revised the manuscript. KW: Collected the samples and performed the analysis. All authors read and approved the final manuscript.
